# The Potential of GPT-4 as a Support Tool for Pharmacists: Analytical Study Using the Japanese National Examination for Pharmacists

**DOI:** 10.2196/48452

**Published:** 2023-10-30

**Authors:** Yuki Kunitsu

**Affiliations:** 1 Department of Pharmacy Shiga University of Medical Science Hospital Otsu, Shiga Japan

**Keywords:** natural language processing, generative pretrained transformer, GPT-4, ChatGPT, artificial intelligence, AI, chatbot, pharmacy, pharmacist

## Abstract

**Background:**

The advancement of artificial intelligence (AI), as well as machine learning, has led to its application in various industries, including health care. AI chatbots, such as GPT-4, developed by OpenAI, have demonstrated potential in supporting health care professionals by providing medical information, answering examination questions, and assisting in medical education. However, the applicability of GPT-4 in the field of pharmacy remains unexplored.

**Objective:**

This study aimed to evaluate GPT-4’s ability to answer questions from the Japanese National Examination for Pharmacists (JNEP) and assess its potential as a support tool for pharmacists in their daily practice.

**Methods:**

The question texts and answer choices from the 107th and 108th JNEP, held in February 2022 and February 2023, were input into GPT-4. As GPT-4 cannot process diagrams, questions that included diagram interpretation were not analyzed and were initially given a score of 0. The correct answer rates were calculated and compared with the passing criteria of each examination to evaluate GPT-4’s performance.

**Results:**

For the 107th and 108th JNEP, GPT-4 achieved an accuracy rate of 64.5% (222/344) and 62.9% (217/345), respectively, for all questions. When considering only the questions that GPT-4 could answer, the accuracy rates increased to 78.2% (222/284) and 75.3% (217/287), respectively. The accuracy rates tended to be lower for physics, chemistry, and calculation questions.

**Conclusions:**

Although GPT-4 demonstrated the potential to answer questions from the JNEP and support pharmacists’ capabilities, it also showed limitations in handling highly specialized questions, calculation questions, and questions requiring diagram recognition. Further evaluation is necessary to explore its applicability in real-world clinical settings, considering the complexities of patient scenarios and collaboration with health care professionals. By addressing these limitations, GPT-4 could become a more reliable tool for pharmacists in their daily practice.

## Introduction

The development of artificial intelligence (AI), as well as machine learning, has led to its application in many industries and fields. AI is increasingly being used in the medical field, for example, to diagnose diseases through diagnostic imaging and to analyze medical records using natural language processing technology [1-3]. More recently, AI chatbots (also known as interactive AI) have been invented and are being used in medicine to automatically converse with human text and voice input [4-6]. As AI chatbots, ChatGPT (GPT-3.5) and GPT-4, released by OpenAI, have high natural language processing capabilities. They are large language models (LLMs) capable of analyzing vast amounts of text data, extracting relevant information, understanding semantic relationships, and generating contextually appropriate responses. Furthermore, it has been reported that ChatGPT and GPT-4 could correctly answer questions on the United States Medical Licensing Examination (USMLE) [7,8] and the Japanese National Medical Licensing Examination [9] at a passing level, despite not having specialized in a particular field of study. Therefore, it is expected to be applied to health care education and to support physicians regarding diagnosis and treatment decisions [10-12]. Despite these promising applications of LLMs, their utility in the context of health care education, particularly in the training and support of health care professionals, such as pharmacists, has yet to be extensively studied. Considering the critical role of pharmacists in patient care and the rapidly evolving landscape of pharmacy practice, evaluating the performance of AI tools like ChatGPT and GPT-4 in addressing pharmacy-related queries is of paramount importance. Pharmacists play a crucial role in the medical field, encompassing a wide range of responsibilities. They are responsible for managing medications, counseling patients on their proper use, providing drug information to both medical staff and patients, and offering suggestions for patient drug treatment plans. In Japan, there have been efforts to use natural language processing AI to manage cases of inquiries about drug information and pharmacological interventions [13]. However, there have been no reports on using widely available AI chatbots. Currently, there is no report on whether AI chatbots can be used in pharmacists’ work, such as drug information and treatment suggestions. Given the potential benefits of AI in supporting health care professionals and the increasing reliance on technology in health care education, there is an emergent need to assess the performance and viability of AI tools like ChatGPT and GPT-4 in these contexts. In this study, the ability of GPT-4 to answer questions on the Japanese National Examination for Pharmacists (JNEP) was evaluated to examine how this AI chatbot can be used as a tool to support pharmacists’ capabilities.

## Methods

### GPT-4

In this study, GPT-4, the latest LLM released as of April 2023, when the study was conducted, was used among the large-scale language models developed by OpenAI. The system is based on the Generative Pretrained Transformer (GPT) architecture and leverages the transformer model. The core concept of GPT-3 was based on a transformer model with 175 billion parameters [14]. Although the exact number of parameters in GPT-3.5 and GPT-4 were not publicly disclosed, it has been adjusted through reinforcement learning from human feedback. GPT-4 was reported to have improved accuracy in its responses compared to GPT-3.5 [15,16]. It is important to note that the model is not specifically fine-tuned for each task but is designed to perform well across a wide range of natural language processing tasks, including question answering, summarization, and dialog generation. At the time used in this study, GPT-4 had accumulated information through September 2021.

### Japanese National Examination for Pharmacists (JNEP)

JNEP is held once a year, and the question texts and answers are published by the Japanese Ministry of Health, Labour, and Welfare. JNEP has undergone several changes in format and content, reflecting the evolving role of pharmacists in health care. These changes have resulted in an increased emphasis on practical skills and knowledge, such as applying pharmaceutical knowledge in a clinical context and understanding relevant laws and ethical considerations. The JNEP criteria are revised approximately every 4 years, with the latest change having occurred at the 106th JNEP in 2020. Because GPT-4 used in this study has information until September 2021, the 107th and 108th JNEPs held in February 2022 and February 2023, respectively, were used.

Each examination consists of 345 questions, divided into 3 blocks: 90 essential questions, 105 pharmacy theory questions, and 150 practical pharmacy questions. The essential questions are questions that examine the basic knowledge required for pharmacists. The pharmacy theory questions are based on the theoretical knowledge necessary to evaluate and solve common problems encountered in pharmacists’ practice. The practical pharmacy questions are designed to assess basic, practical, and general knowledge for solving common problems in health care and public health. The practical pharmacy questions are often based on practical and clinical cases. Each question is from one of the following 9 areas: physics, chemistry, biology, hygiene, pharmacology, pharmaceutics, pathobiology, regulations, and practice. All questions are in the form of multiple-choice answers; however, each question has 1 or 2 correct answers, and if 2 correct answers are chosen, the answer must be complete to be considered correct. In addition to questions in which the examinee must answer correctly or incorrectly from each field, there are questions in which the examinee must perform calculations based on the conditions of the question as well as questions related to drug therapy in the presented case. The calculation questions require examinees to perform calculations from given conditions, and the results are chosen from a list of answer choices. All questions are written in Japanese. The passing criteria for the 107th JNEP included achieving a minimum of 70% correct answers in essential questions, a minimum of 30% correct answers in each area, a minimum of 217 (62.9%) correct answers overall, and a limit of 2 of the several contraindicated choices. Contraindicated choices, in this context, refer to certain choices within the questions that, if selected, could be ethically incorrect, potentially violate laws, or pose significant risks causing harm to patients and public health. Because information on contraindications was not disclosed by the question authors, it was decided to exclude them from the criteria used for evaluation. The passing criteria for the 108th JNEP included 70% or higher correct answers in essential questions, 30% or higher correct answers in each area, 235 (68.1%) or more correct answers overall, and no more than 2 contraindicated choices. In recent years, the pass rate for JNEP has averaged around 70%, with the pass rates for the 107th and 108th JNEPs standing at 68.0% (9607/14,124) and 69.0% (9602/13,915), respectively.

### Data Analysis

**Figure 1:**
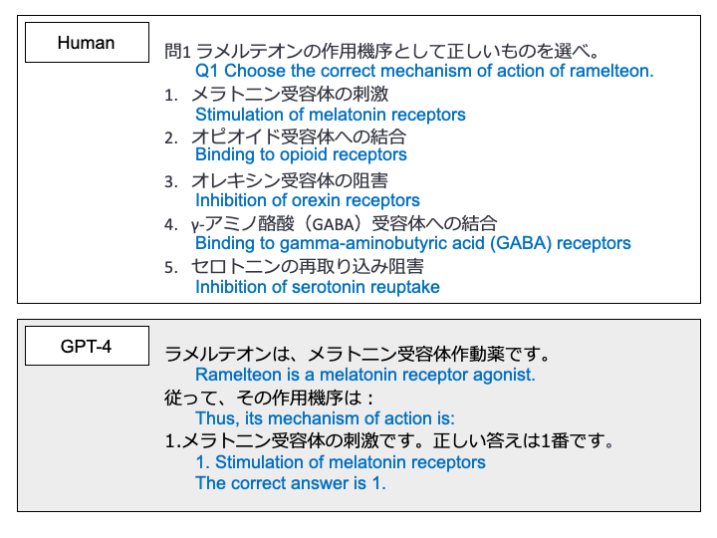
The question input and GPT-4’s response. The question is a sample question. Blue letters are English translations.

Question texts and answer choices of the target JNEP were entered into GPT-4, and the selected choices were tabulated as GPT-4’s answers. The question text was input into GPT-4 in Japanese. Figure 1 shows an image of the question input to GPT-4 and its response.

In the 107th JNEP, there was 1 question for which the answer was undefined, and it was excluded from the list of questions to analyze. During the study, GPT-4 did not allow the input of diagrams as information; therefore, all questions that required reading diagrams to come up with answers were excluded from the answer set. All questions that were not answered were given a score of 0, and the accuracy rate was calculated and compared with the passing criteria. In addition, as a subanalysis, the accuracy rate was calculated by excluding questions that GPT-4 could not answer and then was compared to the passing criteria. Moreover, the accuracy rates were compared according to the question content—whether they were multiple-choice, calculation, or case study questions. For each comparison, the Pearson chi-square test was performed using JMP Pro 16 (SAS Institute Inc).

### Ethics Approval

An ethics approval did not apply to this study. It should be noted that the examination questions and answers used in this study were originally produced and copyrighted by the Ministry of Health, Labour, and Welfare of Japan. These materials are publicly available and used for the purpose of academic research in this study. Any copyrights about the exam content belong to the Ministry of Health, Labour, and Welfare of Japan, and this study did not infringe upon these rights.

## Results

The results of the 107th and 108th JNEPs by GPT-4 are shown in Tables 1 and 2.

For the 107th JNEP, 284 (82.6%) questions were available for input into GPT-4, of which 222 were answered correctly by GPT-4, for its accuracy rate of 64.5% (222/344). GPT-4 could not answer 60 questions that required reading diagrams to come up with answers. In terms of question type, the accuracy rate for essential questions was 72.2% (65/90), the accuracy rate for the pharmacy theory questions was 48.6% (51/105), and the accuracy rate for the practical pharmacy questions was 71.1% (106/149). The accuracy rates for all questions exceeded the passing criteria. However, only 20% (1/5) of the essential questions in chemistry were answerable, which was below the passing criteria. Only for questions that GPT-4 could answer, its accuracy rate for all questions was 78.2% (222/284), meeting all passing criteria.

**Table 1:** The results of the 107th Japanese National Examination for Pharmacists (JNEP) by GPT-4.

JNEP questions			All questions, n		Questions answerable by GPT-4, n		Correct answers, n		Accuracy rate in all questions (%)		Accuracy rate in answerable questions (%)		Passing criteria (%)
**Essential questions**													
	Total	90		76		65		72.2		85.5		≥70	
	Physics	5		4		2		40		50		≥30	
	Chemistry	5		1		1		20		100		≥30	
	Biology	5		2		2		40		100		≥30	
	Hygiene	10		9		9		90		100		≥30	
	Pharmacology	15		15		15		100		100		≥30	
	Pharmaceutics	15		12		8		53.3		66.7		≥30	
	Pathobiology	15		14		13		86.7		92.9		≥30	
	Regulations	10		9		5		50		55.6		≥30	
	Practice	10		10		10		100		100		≥30	
**Pharmacy theory questions**													
	Total	105		74		51		48.6		68.9		—^a^	
	Physics	10		8		3		30		37.5		—	
	Chemistry	10		2		1		10		50		—	
	Biology	10		5		4		40		80		—	
	Hygiene	20		14		8		40		57.1		—	
	Pharmacology	15		14		12		80		85.7		—	
	Pharmaceutics	15		8		4		26.7		50		—	
	Pathobiology	15		14		13		86.7		92.9		—	
	Regulations	10		9		6		60		66.7		—	
	Practice	0		0		0		—		—		—	
**Practical pharmacy questions**													
	Total	149		134		106		71.1		79.1		—	
	Physics	5		4		3		60		75		—	
	Chemistry	5		1		1		20		100		—	
	Biology	5		3		3		60		100		—	
	Hygiene	10		6		6		60		100		—	
	Pharmacology	10		10		10		100		100		—	
	Pharmaceutics	10		9		7		70		77.8		—	
	Pathobiology	10		10		8		80		80		—	
	Regulations	10		10		8		80		80		—	
	Practice	84		81		60		71.4		74.1		—	
**Total questions**													
	Total	344		284		222		64.5		78.2		≥62.9	
	Physics	20		16		8		40		50		—	
	Chemistry	20		4		3		15		75		—	
	Biology	20		10		9		45		90		—	
	Hygiene	40		29		23		57.5		79.3		—	
	Pharmacology	40		39		37		92.5		94.9		—	
	Pharmaceutics	40		29		19		47.5		65.5		—	
	Pathobiology	40		38		34		85		89.5		—	
	Regulations	30		28		19		63.3		67.9		—	
	Practice	94		91		70		74.5		76.9		—	

^a^Not applicable.

**Table 2:** The results of the 108th Japanese National Examination for Pharmacists (JNEP) by GPT-4.

JNEP questions			All questions, n		Questions answerable by GPT-4, n		Correct answers, n		Accuracy rate in all questions (%)		Accuracy rate in answerable questions (%)		Passing criteria (%)
**Essential questions**													
	Total	90		79		65		72.2		82.3		≥70	
	Physics	5		3		1		20		33.3		≥30	
	Chemistry	5		2		1		20		50		≥30	
	Biology	5		2		2		40		100		≥30	
	Hygiene	10		9		8		80		88.9		≥30	
	Pharmacology	15		14		12		80		85.7		≥30	
	Pharmaceutics	15		14		13		86.7		92.9		≥30	
	Pathobiology	15		15		12		80		80		≥30	
	Regulations	10		10		9		90		90		≥30	
	Practice	10		10		7		70		70		≥30	
**Pharmacy theory questions**													
	Total	105		78		58		55.2		74.4		—^a^	
	Physics	10		9		6		60		66.7		—	
	Chemistry	10		0		0		0		—		—	
	Biology	10		6		6		60		100		—	
	Hygiene	20		14		10		50		71.4		—	
	Pharmacology	17		15		11		64.7		73.3		—	
	Pharmaceutics	15		12		8		53.3		66.7		—	
	Pathobiology	13		12		10		76.9		83.3		—	
	Regulations	10		10		7		70		70		—	
	Practice	0		0		0		—		—		—	
**Practical pharmacy questions**													
	Total	150		131		94		62.7		71.8		—	
	Physics	5		3		2		40		66.7		—	
	Chemistry	10		2		1		10		50		—	
	Biology	0		0		0		—		—		—	
	Hygiene	10		8		8		80		100		—	
	Pharmacology	10		9		6		60		66.7		—	
	Pharmaceutics	10		8		5		50		62.5		—	
	Pathobiology	10		10		7		70		70		—	
	Regulations	10		10		6		60		60		—	
	Practice	85		81		59		69.4		72.8		—	
**Total questions**													
	Total	345		288		217		62.9		75.3		≥68.1	
	Physics	20		15		9		45		60		—	
	Chemistry	25		4		2		8		50		—	
	Biology	15		8		8		53.3		100		—	
	Hygiene	40		31		26		65		83.9		—	
	Pharmacology	42		38		29		69.0		76.3		—	
	Pharmaceutics	40		34		26		65		76.5		—	
	Pathobiology	38		37		29		76.3		78.4		—	
	Regulations	30		30		22		73.3		73.3		—	
	Practice	95		91		66		69.5		72.5		—	

^a^Not applicable.

For the 108th JNEP, 288 (83.5%) questions could be input into GPT-4, of which 217 were answered correctly by GPT-4, for its accuracy rate of 62.9% (217/345). GPT-4 could not answer 57 questions, as it required reading diagrams to come up with answers. In terms of question type, the accuracy rate for essential questions was 72.2% (65/90), the accuracy rate for the pharmacy theory questions was 55.2% (58/105), and the accuracy rate for the practical pharmacy questions was 62.7% (94/150). The accuracy rates for all questions and for the essential questions in physics and chemistry were below the passing criteria. Only for questions that GPT-4 could answer, its accuracy rate for all questions was 75.3% (217/288), meeting all passing criteria. Therefore, the accuracy rate for the questions that could be input into GPT-4 for the 107th and 108th JNEP met the passing criteria.

Table 3 shows GPT-4’s accuracy rate across all JNEP questions according to the question type, field, and content, as well as the number of answers. Significant differences in GPT-4’s accuracy rates were observed among the question types (*P*<.001), fields (*P*<.001), and whether or not the question was a calculation question (*P=*.003).

**Table 3:** GPT-4’s accuracy rate in Japanese National Examination for Pharmacists (JNEP) for all questions, broken down by question type, field, content, and answer count.

Variables			Accuracy rate, % (n/N)							*P* value
			The 107th JNEP		The 108th JNEP		The 107th and 108th JNEPs			
All questions			64.5 (222/344)		62.9 (217/345)		63.7 (439/689)		—^a^	
**Type**										<.001
	Essential questions	72.2 (65/90)		72.2 (65/90)		72.2 (130/180)				
	Pharmacy theory questions	48.6 (51/105)		55.2 (58/105)		51.9 (109/210)				
	Practical pharmacy questions	71.1 (106/149)		62.7 (94/150)		66.9 (200/299)				
**Field**										<.001
	Physics	40 (8/20)		45 (9/20)		42.5 (17/40)				
	Chemistry	15 (3/20)		8 (2/25)		11.1 (5/45)				
	Biology	45 (9/20)		53.3 (8/15)		48.6 (17/35)				
	Hygiene	57.5 (23/40)		65 (26/40)		61.3 (49/80)				
	Pharmacology	92.5 (37/40)		69.0 (29/42)		80.5 (66/82)				
	Pharmaceutics	47.5 (19/40)		65 (26/40)		56.3 (45/80)				
	Pathobiology	85 (34/40)		76.3 (29/38)		80.8 (63/78)				
	Regulations	63.3 (19/30)		73.3 (22/30)		68.3 (41/60)				
	Practice	74.5 (70/94)		69.5 (66/95)		72.0 (136/189)				
**Calculation questions**										.003
	Questions requiring a calculation	37.5 (6/16)		40 (6/15)		38.7 (12/31)				
	Questions not requiring a calculation	65.9 (216/328)		63.9 (211/330)		64.9 (427/658)				
**Case questions**										.27
	Questions in a clinical case	73.0 (100/137)		59.6 (84/141)		66.2 (184/278)				
	Questions not in a clinical case	58.9 (122/207)		65.2 (133/204)		62.0 (255/411)				
**Number of answers**										.63
	1	63.5 (120/189)		62.4 (128/205)		62.9 (248/394)				
	2	65.8 (102/155)		63.6 (89/140)		64.7 (191/295)				

^a^Not applicable.

This result was also obtained from GPT-4’s accuracy rate in the JNEP, specifically for questions that GPT-4 could answer (Table 4).

**Table 4:** GPT-4’s accuracy rate in Japanese National Examination for Pharmacists (JNEP) for questions that GPT-4 could answer, broken down by question type, field, content, and answer count.

Variables		Accuracy rate (%)				*P* value
		The 107th JNEP	The 108th JNEP	The 107th and 108th JNEPs		
All questions		78.2 (222/284)	75.3 (217/288)	76.7 (439/572)	—^a^	
**Type**						.03
	Essential questions	85.5 (65/76)	82.3 (65/79)	83.9 (130/155)		
	Pharmacy theory questions	68.9 (51/74)	74.4 (58/78)	71.7 (109/152)		
	Practical pharmacy questions	79.1 (106/134)	71.8 (94/131)	75.5 (200/265)		
**Field**						.006
	Physics	50 (8/16)	60 (9/15)	54.8 (17/31)		
	Chemistry	75 (3/4)	50 (2/4)	62.5 (5/8)		
	Biology	90 (9/10)	100 (8/8)	94.4 (17/18)		
	Hygiene	79.3 (23/29)	83.9 (26/31)	81.7 (49/60)		
	Pharmacology	94.9 (37/39)	76.3 (29/38)	85.7 (66/77)		
	Pharmaceutics	65.5 (19/29)	76.5 (26/34)	71.4 (45/63)		
	Pathobiology	89.5 (34/38)	78.4 (29/37)	84 (63/75)		
	Regulations	67.9 (19/28)	73.3 (22/30)	70.7 (41/58)		
	Practice	76.9 (70/91)	72.5 (66/91)	74.7 (136/182)		
**Calculation questions**						<.001
	Questions requiring a calculation	42.9 (6/14)	42.9 (6/14)	42.9 (12/28)		
	Questions not requiring a calculation	80 (216/270)	77.0 (211/274)	78.5 (427/544)		
**Case questions**						.34
	Questions in a clinical case	80 (100/125)	69.4 (84/121)	74.8 (184/246)		
	Questions not in a clinical case	76.7 (122/159)	79.6 (133/167)	78.2 (255/326)		
**Number of answers**						.12
	1	81.1 (120/148)	77.6 (128/165)	79.2 (248/313)		
	2	75 (102/136)	72.4 (89/123)	73.7 (191/259)		

^a^Not applicable.

## Discussion

### Principal Findings

The results of inputting the 107th and 108th JNEP questions into GPT-4 showed that GPT-4 failed to meet some passing criteria. However, only for questions that GPT-4 could answer, its accuracy rate met all the passing criteria. In the past, LLMs have demonstrated the ability to answer several professional examinations at a passing level. For example, ChatGPT has been reported to be capable of answering questions of the law school and business management course examinations [17] and the final exam for the Master of Business Administration field [18] at a passing level. Furthermore, for the medical field, it has been reported that ChatGPT’s score on the USMLE is equivalent to the passing score of third-year medical students [7] or close to the passing standard [8]. In Japan’s National Medical Practitioners Qualifying Examination, the accuracy rate was also reported to be 55.0% [19]. The results of this study showed a higher accuracy rate than ChatGPT’s performance in medical examinations, as reported in previous studies. The main reason for this difference is thought to be the distinct LLM used and the varying knowledge requirements for physicians and pharmacists. The LLM used in this study was GPT-4, which was released on March 14, 2023. It is said to have had a more complex neural network and larger training data set than the older models [20], which may have led to the results of this study. Kasai et al [9] reported that GPT-4 achieves the best performance on Japan’s National Medical Practitioners Qualifying Examination questions compared to ChatGPT, ChatGPT-EN, and GPT-3, and it passed the exams of all 6 years [9]. In the study by Kasai et al [9] and this study, the questions were entered in Japanese, indicating that GPT-4 is highly effective in decoding content and providing accurate answers without translation into English. The GPT-4 Technical Report [21] reported different accuracy rates for questions in English and Japanese (85.5% vs 79.9%). Therefore, it is suggested that higher accuracy rates may be obtained by translating questions into English and then inputting them into GPT-4.

Although GPT-4 is not specifically trained or specialized in any particular field of study, it has demonstrated a certain level of ability to respond to questions in each of these areas. However, the accuracy rate varied depending on the field of questions. When limited to questions that GPT-4 could answer, accuracy rates tended to be higher for biology and pharmacology questions and lower for physics and chemistry. Nisar et al [22] reported the results of the ChatGPT test on pharmacology for undergraduate students, which showed that they adequately answered various questions on drug’s pharmacokinetics, mechanism of action, clinical uses, adverse effect, contraindications, and drug-drug interactions [22]. On the other hand, in physics questions, GPT-4 often answered incorrectly to questions about analysis techniques, such as liquid chromatography and electrophoresis, as well as questions about purity tests and determination methods, which are described in the Japanese Pharmacopoeia [23]. Although analysis methods and the Japanese Pharmacopoeia in English can be searched on the internet [23], it is a highly technical field, and GPT-4 may not have been adequately studied. In Antaki et al’s [24] report of the evaluation of ChatGPT answers to ophthalmology questions, the results were good for general medicine but not for highly specialized fields, such as neuro-ophthalmology and ocular pathology. Therefore, it is expected that GPT-4 would perform lower in highly specialized areas due to inadequate learning. Many of the chemistry questions included diagrams of chemical structures, and only 16% (8/50) of chemistry questions could be input into GPT-4. Therefore, it is impossible to clarify the performance of GPT-4 with the chemistry field from this result.

In this survey, there were not only simple correct or incorrect questions about events but also many questions in which a case was presented and the question was about pharmacotherapy for the case. The accuracy rate of the case questions that GPT-4 could answer was 74.8%, which was as high as the percentage for all questions except for the case questions. This indicates that GPT-4 could be used by pharmacists to support their pharmacotherapy practice in clinical settings. However, in discussing the limitations of GPT-4 in real-world pharmacy practice, several factors should be considered. Although GPT-4 demonstrated strong performance on standardized exam questions, its effectiveness in handling diverse clinical scenarios and patient-specific factors may be limited. This is primarily due to the challenge of processing a wide range of patient information that extends beyond the scope of exam questions. In real-world clinical settings, patient data include detailed medical history, medication history, laboratory data, and allergy information, which change with time. It is unclear whether GPT-4 can accurately process such diverse information. Another important consideration is the lack of communication skills with other health care professionals. In real clinical practice, pharmacists collaborate and exchange information with various members of the health care team. However, GPT-4 cannot mimic this collaborative communication with other professionals. It was reported that ChatGPT lacks thoughtful reasoning like humans [25] and cannot evaluate information critically [26]. Consequently, the utility of GPT-4 in team-based health care provision may be limited. By recognizing and addressing these limitations, a more comprehensive evaluation of GPT-4’s practical applicability in clinical settings can be achieved. It is essential to acknowledge that GPT-4’s effectiveness in handling the complexities of real-world clinical practice, including diverse patient scenarios and collaboration with other health care professionals, needs further consideration and exploration. In addition, it is important to note that GPT-4, as it currently stands, is not compliant with patient privacy information [26].

### Limitations

It is important to note that the accuracy rate of GPT-4 was not 100%, and caution is needed regarding ethical issues related to the input of patients’ personal information [26]. It is reported that ChatGPT is prone to a phenomenon known as “hallucination,” which involves the generation of scientifically false content that appears sound to nonexperts [26]. Therefore, it is risky to rely completely on the generated content.

In addition, the accuracy rate for questions for which the participants were required to perform calculations under the indicated conditions was low (42.9%). In some cases, the results were incorrect due to the omission of values with different units, and in other cases, the results were correct; however, GPT-4 made a mistake in selecting the option with the closest value. In a previous report [17], it has been noted that there were surprisingly erroneous answers to the calculation questions, and the answers to the calculation questions were considered unreliable. Furthermore, questions that included diagrams could not be entered in this survey and were excluded from the answers, but diagram recognition is essential to a pharmacist’s ability to infer drug characteristics from the structural formula of a substance or to predict drug changes from chemical reaction formulas. These limitations should be considered when using GPT-4 in clinical practice. It is expected that in the future, LLMs will be developed to be capable of recognizing diagrams and photos as information. Furthermore, although the level of knowledge required of pharmacists was assessed by having them answer the JNEP questions using GPT-4, this may not entirely reflect the knowledge and suggestions that pharmacists are required to provide in clinical settings. GPT-4 does not have an inherent knowledge of which answers are right or wrong but rather generates responses based on patterns and information present in its training data. Therefore, it cannot provide responses beyond the information present in its training data available on the web. However, it is important to note that pharmacists may often face questions and scenarios that are not readily available on the internet. In addition to the limitations discussed above, it is important to acknowledge that the GPT-4 model used in this study was pretrained until September 2021 and does not have access to the internet or other resources beyond that date. Given the rapidly changing nature of fields like pharmacy, which sees the introduction of new medications annually and the release of updated treatment guidelines every few years, it is essential to recognize that GPT-4 may not be up to date with the latest information. This study provides insights into GPT-4’s capabilities within its training data timeframe, and therefore, caution should be exercised when applying its results to real-world clinical practice, and reliance on the most current sources and specialized knowledge is necessary. To evaluate whether GPT-4 can be used as an auxiliary tool for pharmacist work in the future, verification using more detailed work data sets (eg, patient counseling, records of inquiries from physicians, drug interaction analysis, and examples of questionable prescriptions) is required.

### Conclusions

In conclusion, GPT-4 showed that some passing criteria were not met in terms of the accuracy rate for all JNEP questions, but the accuracy rates for the questions that GPT-4 could answer met all of the passing criteria. Nevertheless, recognizing the limitations of the current GPT-4 model is crucial, particularly in terms of its performance in answering highly specialized questions, calculation questions, and questions requiring diagram recognition. Furthermore, exploring the practical applicability of GPT-4 in real-world clinical settings is essential by evaluating its performance on more detailed work data sets (eg, patient counseling, records of inquiries from physicians, drug interaction analysis, and examples of questionable prescriptions). By addressing these limitations and validating its performance in a broader range of tasks, GPT-4 could become a more reliable and effective tool for pharmacists in their day-to-day practice.

## Appendix

Multimedia Appendix 1 GPT-4’s answers, along with the question type, field, and content, as well as the number of answers for each question from the 107th and 108th Japanese National Examination for Pharmacists (JNEP).

## Abbreviations

AI: artificial intelligence
GPT: Generative Pretrained Transformer
JNEP: Japanese National Examination for Pharmacists
LLM: large language model
USMLE: United States Medical Licensing Examination

## Data Availability

All data analyzed during this study are included in Multimedia Appendix 1.
